# A Novel Method of Path Planning for an Intelligent Agent Based on an Improved RRT* Called KDB-RRT*

**DOI:** 10.3390/s25247545

**Published:** 2025-12-12

**Authors:** Wenqing Wei, Kun Wei, Jianhui Zhang

**Affiliations:** 1 School of Automobile and Transportation, Tianjin University of Technology and Education, Tianjin 300222, China; eeinqing@163.com (W.W.); xiaohui009819@163.com (J.Z.); 2 National and Local Joint Engineering Research Center for Intelligent Vehicle Road Collaboration and Safety Technology, Tianjin 300222, China

**Keywords:** path planning, RRT*, KD-tree, Sigmoid function, Douglas–Peucker algorithm

## Abstract

To address challenges in agent path planning within complex environments—particularly slow convergence speed, high path redundancy, and insufficient smoothness—this paper proposes KDB-RRT*, a novel algorithm built upon RRT.* This method integrates a bidirectional search strategy with a three-layer optimization framework: ① accelerated node retrieval via KD-tree indexing to reduce computational complexity; ② enhanced exploration efficiency through goal-biased dynamic circle sampling and a bidirectional gravitational field guidance model, coupled with adaptive step size adjustment using a Sigmoid function for directional expansion and obstacle avoidance; and ③ trajectory optimization employing DP algorithm pruning and cubic B-spline smoothing to generate curvature-continuous paths. Additionally, a multi-level collision detection framework integrating Separating Axis Theorem (SAT) pre-judgment, R-tree spatial indexing, and active obstacle avoidance strategies is incorporated, ensuring robust collision resistance. Extensive experiments in complex environments (Z-shaped map, loop-shaped map, and multi-obstacle settings) demonstrate KDB-RRT’s superiority over state-of-the-art methods (Optimized RRT*, RRT*-Connect, and Informed-RRT*), reducing average planning time by up to 97.9%, shortening path length by 5.5–21.4%, and decreasing inflection points by 40–90.5%. Finally, the feasibility of the algorithm’s practical application was further verified based on the ROS platform. The research results provide a new method for efficient path planning of intelligent agents in unstructured environments, and its three-layer optimization framework has important reference value for mobile robot navigation systems.

## 1. Introduction

The rapid advancement of artificial intelligence has led to the widespread application of agents as flagship products across diverse fields. Path planning technology [1] stands as a core component of agent research, directly determining the quality of an agent’s movement trajectory and consequently impacting its task execution efficiency and success rate. Therefore, research into autonomous path planning for agents holds significant importance for the development of the AI industry.

Current path planning methodologies primarily include the A* algorithm [2], artificial potential field method [3], randomized sampling algorithms [4], ant colony optimization [5], and genetic algorithms [6]. In recent decades, the Rapidly exploring Random Tree (RRT) algorithm [7], based on randomized search strategies, has gained extensive adoption in agent path planning due to its probabilistic completeness and scalability. However, it suffers from drawbacks such as excessive planning time, high path cost, and low path quality, failing to guarantee planning speed and efficiency. To address these limitations, researchers globally have proposed various RRT variants. Karaman and Frazzoli [8] introduced the asymptotically optimal RRT* algorithm, which enhances path quality through parent node reselection and rewiring, converging towards the asymptotic optimum, albeit at the expense of higher computational cost, relatively lower efficiency, and longer search times. Akgun and Stilman [9] proposed the Bi-RRT algorithm, employing bidirectional expansion by growing trees simultaneously from the start and goal points until connection, improving search efficiency and stability by balancing tree growth, yet it still incurs significant computation and may be constrained by complex environments. LaValle and Kuffner [10] developed the RRT-Connect algorithm, combining RRT with a greedy heuristic by alternately growing trees from start and goal configurations; although it offers high search efficiency, its path quality may be inferior to RRT*, and it can become trapped in local optima in certain scenarios. Xie Gaoyang et al. [11] presented an improved RRT algorithm matching vehicle speed to expansion step size, offering a novel solution for path planning of unmanned target vehicles at different speeds, though path quality may degrade in complex situations. Lim Subin et al. [12] proposed the Ex-RRT* algorithm, incorporating a cost function based on the distance from each node to the nearest obstacle to ensure adequate clearance, thereby enhancing path safety. Gu Zilv et al. [13] introduced a goal-biased node selection strategy, reducing algorithm randomness, but convergence efficiency remains slow in complex environments. Ma Xiaoqun et al. [14] developed an IRRT*-Connect algorithm dependent on environmental complexity, improving adaptability to complex settings, yet the issue of low computational efficiency persists. Li Wenjun et al. [15] addressed problems in RRT* such as excessive redundant points, jagged paths, and proximity to obstacles in multi-obstacle environments with their Safe-Smooth RRT* algorithm, enhancing path smoothness, although performance may decline as obstacle density increases. Liu Wenguang et al. [16] proposed an RRT variant with parent reselection within a constrained range, but search times can become lengthy in multi-obstacle environments.

To address the issues of high computational cost, low search efficiency, and suboptimal path quality in RRT, this paper proposes a KD-tree-based Goal-biased Dual RRT* (KDB-RRT*) algorithm. Leveraging the asymptotic optimality of RRT* and incorporating a bidirectional search strategy, KDB-RRT* achieves performance breakthroughs through a three-layer optimization framework: (1) Spatial Indexing Acceleration: utilizing a KD-tree to expedite nearest-neighbor queries, significantly reducing computational complexity; (2) Guided Sampling and Expansion: employing a dynamic circular sampling strategy and bidirectional target-biased gravitational field guidance, combined with adaptive step size adjustment based on the Sigmoid function, enhancing directional bias during path expansion and obstacle avoidance capability in complex regions; (3) Path Morphology Optimization: applying the Douglas–Peucker (DP) algorithm for path pruning and employing cubic B-spline curves to achieve curvature-continuous smoothing, optimizing both path length and smoothness. And a multi-level collision detection framework integrating Separating Axis Theorem (SAT) pre-judgment, R-tree spatial indexing, and active obstacle avoidance strategies is incorporated, ensuring robust collision resistance. Comparative experiments against Optimized RRT*, RRT-Connect, and Informed-RRT* demonstrate that KDB-RRT* significantly enhances planning efficiency and trajectory quality while preserving probabilistic completeness.

## 2. Related Work

### 2.1. Basic Principle of RRT* Algorithm

**Figure 1:**
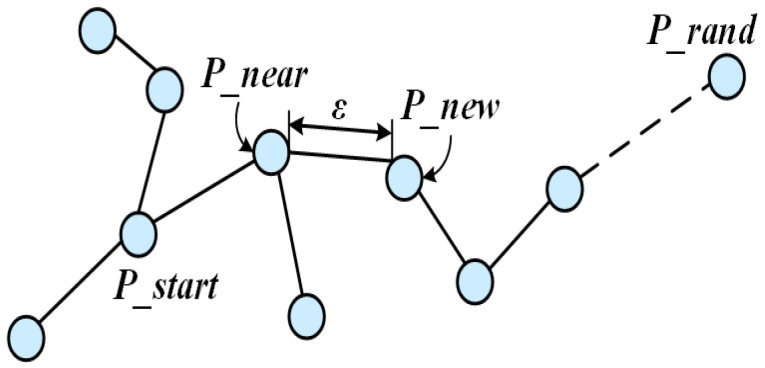
Basic principle diagram of RRT.

Path expansion in the conventional RRT algorithm is guided by random sampling points. Its core mechanism explores potential paths through stochastic spatial growth of the path tree. As illustrated in Figure 1, the algorithm first identifies a start node $P_{\mathrm{start}}$ in free space and generates a random sample point $P_{\mathrm{rand}}$. It then locates the nearest node $P_{\mathrm{near}}$ to $P_{\mathrm{rand}}$ within the existing tree and attempts to connect $P_{\mathrm{near}}$ and $P_{\mathrm{rand}}$. If the connecting segment is collision-free, a new node $P_{\mathrm{new}}$ is generated by extending a fixed step size ε in this direction and added to the tree. This process repeats until the goal node (or a node within its proximity) is incorporated. The final path is generated by backtracking from $P_{\mathrm{goal}}$ to $P_{\mathrm{start}}$.

**Figure 2:**
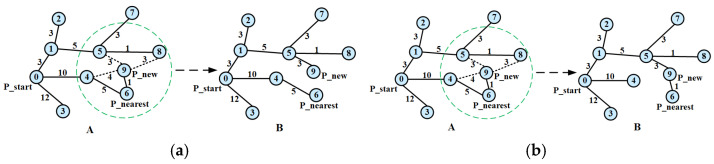
Basic principle diagram of RRT*. (**a**) Stage 1: Re-select Parent Nodes; (**b**) Stage 2: Rewire the random tree.

RRT* achieves asymptotic optimality through dynamic parent-node reselection and path-tree rewiring, comprising two stages:

Stage 1: Parent Node Reselection. As depicted in Figure 2a, after generating $P_{\mathrm{new}}$, a neighborhood ball of radius r (green circle) forms a node set N. The node in N yielding minimal path cost to $P_{\mathrm{new}}$ is selected as its new parent.

Stage 2: Path Tree Rewiring. To further reduce the global path cost, as shown in Figure 2b, a neighborhood ball of radius r (green circle, analogous to Stage 1) delimits the scope of neighboring nodes $P_{i}$ to be checked. Neighboring nodes $P_{i}$ are traversed; if rerouting $P_{i}$ through $P_{\mathrm{new}}$ reduces its path cost, $P_{\mathrm{new}}$ becomes $P_{i}$’s parent. Iterative optimization continues until no further cost reduction is possible.

### 2.2. Basic Principle of B-RRT* Algorithm

**Figure 3:**
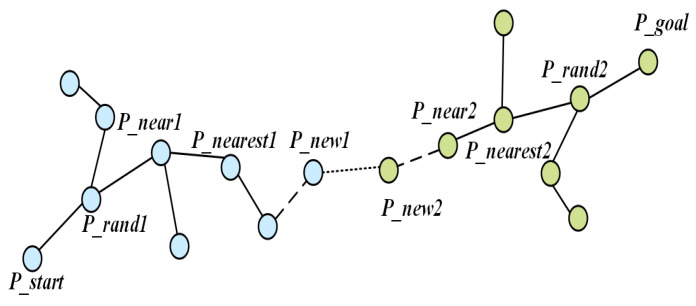
Basic principle diagram of B-RRT*.

The B-RRT* (Bidirectional Rapidly exploring Random Tree Star) algorithm enhances the search efficiency of the RRT* algorithm by incorporating a bidirectional search strategy. Its core concept involves constructing two separate path trees originating from the start and goal configurations, respectively—in Figure 3, the tree rooted at $P_{\mathrm{start}}$ is represented in blue, while the tree rooted at $P_{\mathrm{goal}}$ is shown in green. These trees alternately expand until they connect, as illustrated in Figure 3. This strategy balances the bidirectional search scope, reducing inefficient exploration in a single direction. Particularly in complex environments, it can significantly shorten search time. However, its inherent random sampling nature may still lead to insufficient convergence efficiency, necessitating further optimization through goal-biasing mechanisms.

## 3. Multi-Layer Optimization Architecture of KDB-RRT* Algorithm

Although the B-RRT* algorithm improves search efficiency, each iteration requires traversing all nodes in the search tree and selecting suitable nodes based on Euclidean distance, resulting in substantial computational overhead that severely impacts planning efficiency during iterations. To address this, this paper proposes the KDB-RRT* algorithm. By integrating the bidirectional search framework with multi-dimensional optimization strategies, it constructs a three-layer technical architecture comprising Spatial Indexing Acceleration, Dynamic Sampling Guidance, and Path Morphology Optimization. This architecture significantly enhances path planning efficiency and trajectory quality in complex environments.

### 3.1. KD-Tree Indexing for Nearest-Neighbor Query Optimization

As an efficient multi-dimensional spatial indexing structure, the KD-tree (K-Dimensional Tree) has been widely used in recent years to optimize the computational efficiency of data-intensive algorithms. Its core value lies in significantly reducing computational complexity through spatial partitioning. Chen et al. [17] introduced the KD-tree into the DBSCAN algorithm (KD-DBSCAN). By pre-partitioning datasets to construct neighborhood object sets, redundant computations are avoided, providing a new approach for processing large-scale spatiotemporal data such as GPS trajectories. Xue et al. [18] proposed a single KD-tree-based approximate nearest neighbor algorithm and a multi-KD-tree cross-search algorithm, addressing the bottleneck of iterative computation efficiency in big data scenarios. Sun et al. [19] innovatively integrated the R*-tree with the KD-tree to build a hybrid index, and combined multi-dimensional histograms and hash functions to achieve efficient join queries for Linked Open Data. Xiu et al. [20] proposed the Grid KD-Tree (GKDT) structure, which, at the cost of partial storage space, significantly improved the online point location speed in explicit model predictive control. These studies collectively demonstrate that the KD-tree, leveraging its hierarchical spatial partitioning property, has become a key technology for optimizing the spatiotemporal complexity of algorithms. Its innovative applications have expanded from cluster analysis to data querying and real-time control, with the core contribution being “trading space for time” to effectively address the computational efficiency bottleneck in high-dimensional, large-scale data scenarios. Therefore, to tackle the high computational complexity of node retrieval in the B-RRT* algorithm, this paper intends to introduce the KD-tree (K-Dimensional Tree) structure to achieve fast localization of nearest neighbor nodes.

#### 3.1.1. KD-Tree Organization

**Figure 4:**
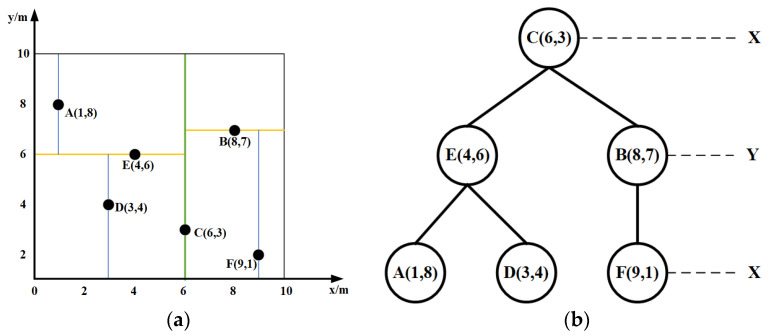
KD-tree organization and 2D space division. (**a**) Construction of KD-tree in 2D space; (**b**) organizational construction of KD-tree.

Consider constructing a KD-Tree for a node set on a random tree T: {A (1,8), B (8,7), C (6,3), D (3,4), E (4,6), F (9,1)}. The construction process (as shown in Figure 4) is as follows:

In Figure 4a, the green vertical line represents the partitioning plane along the *x*-dimension in Step 2, the yellow horizontal line denotes the partitioning plane along the y-dimension in Step 3, and the blue color indicates the subsequent partitioning planes in Step 4, visually illustrating the alternating partitioning axes at different levels.

1. Select Splitting Axis: Calculate the variance for both the *x* and *y* dimensions. Compare the variances and select the dimension with the larger variance as the splitting axis. Using Equation (1), the variances for the *x* and *y* dimensions in this example are calculated as $\sigma_{x}^{2}=7.81$ and $\sigma_{y}^{2}=5.56$, respectively. Since $\sigma_{x}^{2}>\sigma_{y}^{2}$, the x-dimension is chosen as the first splitting axis.
  (1) $$\sigma^{2}=\frac{{\left(x_{1}-\bar{x}\right)}^{2}+{\left(x_{2}-\bar{x}\right)}^{2}+\ldots +{\left(x_{n}-\bar{x}\right)}^{2}}{n}$$
2. Determine Median Point on Axis: Sort the data points based on the selected splitting dimension and choose the median point as the root node. Sorting all points by the x-dimension yields: A (1,8), D (3,4), E (4,6), C (6,3), B (8,7), F (9,1). The median point is C (6,3), which becomes the root node.
3. Determine Left and Right Subtrees: Using the selected splitting axis and the median point as the splitting plane, construct the left and right subtrees. In this example, the left subtree contains {A (1,8), D (3,4), E (4,6)} and the right subtree contains {B (8,7), F (9,1)}.
4. Recurse on Subtrees: Repeat steps (1) to (3) recursively for each subtree until each contains only one data point. Note: The splitting axis is typically selected by cycling through dimensions sequentially. Since the x-dimension was used at the first level, the y-dimension is used at the second level, the x-dimension again at the third level, and so on. In this example, step (4) proceeds as follows:
  - For the left subtree {A, D, E}, select the y-dimension as the splitting axis. The median point on this axis is E (4,6). This splits the subtree into left child A (1,8) and right child D (3,4).
  - For the right subtree {B, F}, select the y-dimension as the splitting axis. Point B (8,7) is chosen as the root node for this subtree (as it has the larger y-coordinate). This splits the subtree into right child F (9,1).

#### 3.1.2. KD-Tree Nearest Neighbor Search

**Figure 5:**
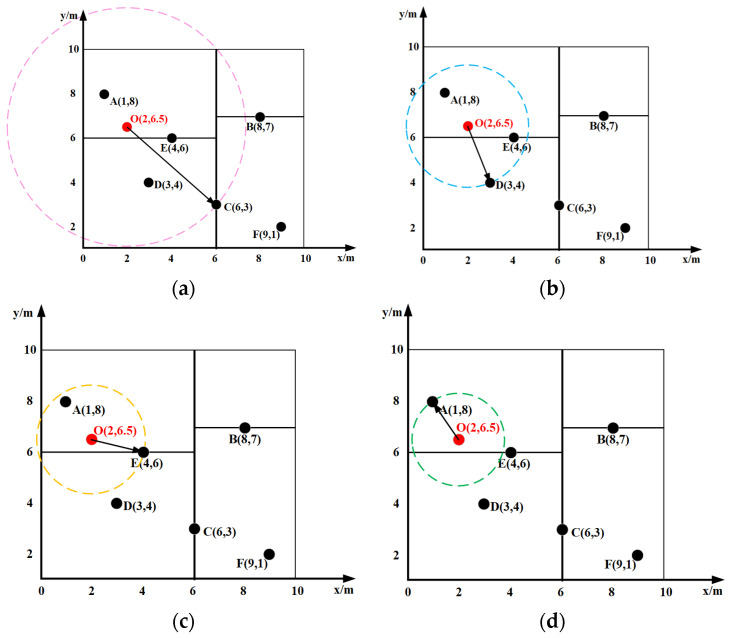
KD-tree nearest neighbor search. (**a**) Take C as the nearest neighbor; (**b**) Update D as the nearest neighbor; (**c**) Update E as the nearest neighbor; (**d**) Update A as the nearest neighbor.

Utilizing the constructed KD-Tree, the process of finding the nearest neighbor node $P_{\mathrm{near}}$ for a query point is as follows (refer to Figure 5):

In Figure 5, red, blue, yellow, and green circles respectively correspond to the search spheres in Steps 1–4, illustrating the iterative refinement of the nearest neighbor during backtracking.

1. Start at the root node C. Assume C is the current nearest neighbor. Define a red search circle centered at O with radius OC (Figure 5a). This circle intersects the hyperplanes *y* = 6 and *y* = 7. Since 6 < 7, search the right subspace of C first.
2. Find E (4,6) in 1. The splitting hyperplane at C is *y* = 6. As O’s y-coordinate is 6.5, proceed to the right subspace, finding D (3,4). The search path is C (6,3) → E (4,6) → D (3,4). Set D as the current nearest neighbor. Define a new (blue) search circle centered at O with radius OD (Figure 5b).
3. Backtrack to E (4,6), and calculate OC. Since OE < OD, update the current nearest neighbor to E (4,6). Define a new (yellow) search circle with radius OE (Figure 5c).
4. The yellow circle intersects the hyperplane *y* = 4. Therefore, search the other subspace of E (4,6) (the left subtree). Calculate OA. Since OA < OC, update the current nearest neighbor to A (1,8) and the distance to OA. Define a new (green) search circle (Figure 5d).
5. Backtrack to the root C (6,3). The green circle does not intersect the hyperplane x = 6, so searching the right subspace of C (6,3) is unnecessary. The search concludes, returning A (1,8) as the nearest neighbor O with distance OA.

Once node A is identified as the nearest neighbor $P_{\mathrm{near}}$ to the query point, it is added to the current tree. The KD-tree is dynamically updated to incorporate A into its structure. Subsequently, new nodes are generated through expansion based on the growth guidance model (Section 3.3) and adaptive step size (Section 3.4). During each iteration, a new random sample point is generated, and an independent nearest-neighbor search is executed using the updated KD-tree.

#### 3.1.3. Complexity Analysis

For a two-dimensional dataset containing n nodes, the time complexity of a single-level partition is $O\left(n\right)$, analyzed as follows:

1. Select Splitting Axis: Cyclic dimension switching (x→y→x→⋯) requires $O\left(1\right)$ time, as the number of dimensions d=2 for 2D path planning) is fixed and independent of the dataset size n.
2. Determine Median Point on Axis: The randomized quickselect algorithm achieves average-case $O\left(n\right)$ complexity, avoiding worst-case $O\left(n^{2}\right)$ performance through randomized pivot selection.
3. Determine Left and Right Subtrees: Distributing the remaining n−1 nodes into left/right subtrees requires $O\left(n\right)$ time.

Thus, the total time for a single-level partition is $O\left(1\right)+O\left(n\right)+O\left(n\right)=O\left(n\right)$.

Let $T\left(n\right)$ denote the time complexity for constructing a KD-tree with n nodes. Since each partition splits the dataset into two roughly equal subsets (size of $\frac{n}{2}$ for each), the recurrence relation is:

(2) $$\begin{array}{l} T\left(n\right)=O\left(n\right)+2\cdot T\left(\frac{n}{2}\right)\\ =O\left(n\right)+2\cdot \left[O\left(\frac{n}{2}\right)+2\cdot T\left(\frac{n}{4}\right)\right]\\ =O\left(n\right)+O\left(n\right)+4\cdot T\left(\frac{n}{4}\right)\\ \vdots \\ =O\left(n\right)+O\left(n\right)+\cdots +O\left(n\right)\\ =O\left(n\log n\right) \end{array}$$

The average-case time complexity for KD-tree construction is therefore $O\left(n\log n\right)$.

In contrast, traditional exhaustive search methods exhibit $O\left(n\right)$ per-query complexity equivalent to the worst-case per-query complexity of KD-tree nearest-neighbor searches. For large n, the per-query complexity of traditional methods ($O\left(n\right)$) significantly exceeds that of KD-tree-based approaches ($O\left(n\log n\right)$). Consequently, KD-trees represent a widely adopted efficient solution for multidimensional search problems.

Efficiency is particularly enhanced when $n\geq 2^{d}$ for d-dimensional data. In this study, agent path planning operates in two-dimensional space (d=2), a condition readily satisfied.

Integrating KD-trees into nearest-neighbor searches during path planning directly reduces the computational bottleneck of RRT-based algorithms. By accelerating the critical “nearest node” retrieval step from $O\left(n\right)$ to $O\left(n\log n\right)$, KD-trees minimize redundant computations, thereby enhancing overall path planning efficiency, especially in cluttered environments with large node sets.

### 3.2. Target-Biased Dynamic Circular Sampling Strategy

Although the KDB-RRT* algorithm incorporating the KD-Tree significantly improves path planning efficiency, it is still unavoidable to prevent the problem of low convergence speed caused by the strong randomness of sampling in complex environments. To address the blindness of uniform sampling, this paper proposes a Dynamic Circle Sampling Strategy with Goal Bias. The dynamic sampling radius $R\left(d\right)$ is defined as:

(3) $$R\left(d\right)=R_{0}\times \left[1-{\left(\frac{d}{D}\right)}^{k}\right]\left(k\geq 1\right)$$

where $R_{0}$ is the initial sampling radius; $d=\left\|P_{i}-P_{\mathrm{goal}}\right\|\left(i=1,2,\ldots \right)$ is the Euclidean distance from the current node to the goal; $D=\left\|P_{start}-P_{goal}\right\|$ is the Euclidean distance from start to goal, used to normalize d; k is a scaling factor controlling the rate of radius change with distance. When d→D (the current node is far from the goal), $R\left(d\right)$ increases to accelerate exploration towards the goal region. When d→0 (the current node is close to the goal), $R\left(d\right)$ decreases to enable localized refinement.

**Figure 6:**
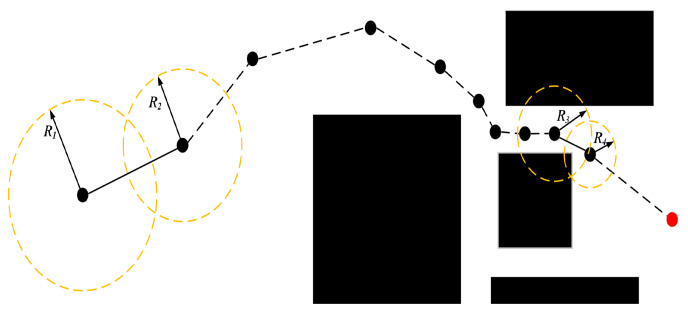
Generation of dynamic circular sampling region.

During iteration, a dynamic sampling region Ω is established centered on the current node $P_{near}$, as defined in Equation (4). The random sample $P_{\mathrm{rand}}$ is constrained within Ω. The dynamic sampling process throughout path planning is depicted in Figure 6.

The dynamic sampling process throughout path planning is depicted in Figure 6, where yellow dashed circles represent the dynamic sampling regions Ω centered on different $P_{near}$; their varying sizes illustrate how $R\left(d\right)$ adapts to the distance between the current node and the goal (larger circles correspond to greater distances, consistent with Equation (3)).

(4) $$\Omega =\left\{P\in {\mathbb{R}}^{2}\left\|P-P_{near}\right\|\leq R\left(d\right)\right\}$$

### 3.3. Bidirectional Growth Guidance Model Based on Potential Field

**Figure 7:**
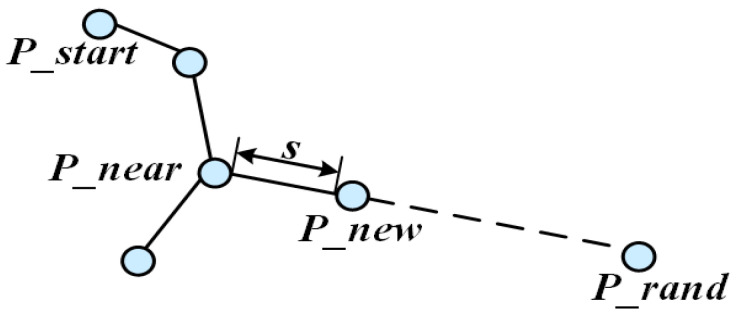
Principal diagram of new node expansion in B-RRT*.

**Figure 8:**
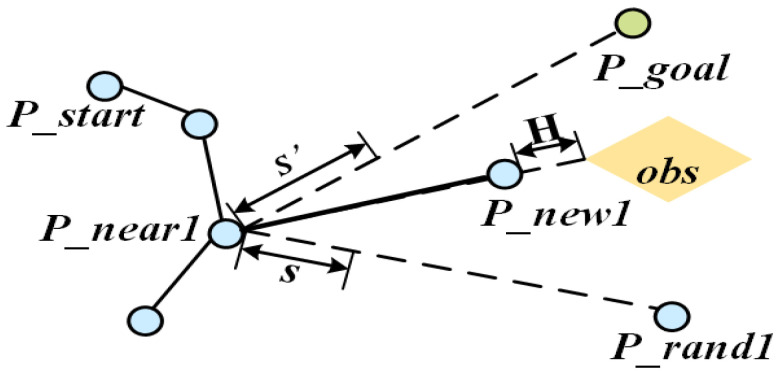
Schematic diagram of new node expansion based on potential field guidance.

In the B-RRT* algorithm, the two trees ($T_{start}$ and $T_{goal}$) grow towards each other’s root ($P_{start}$ or $P_{goal}$) as their respective targets. New node expansion follows Figure 7 and Equation (5):

(5) $$P_{new}=P_{near}+s\cdot \vec{F}$$

where $\vec{F}=\frac{P_{rand}-P_{near}}{\left\|P_{rand}-P_{near}\right\|}$ is a unit vector in a random direction, and s is a fixed step size. However, this expansion is highly random and lacks strong goal directionality. To enhance directional bias, this paper introduces an attractive field-based growth guidance model into the bidirectional search. For $T_{start}$ and $T_{goal}$, attractive vectors are defined:

(6) $$\vec{G_{1}}=\lambda \times \frac{P_{goal}-P_{near}}{\left\|P_{goal}-P_{near}\right\|},\vec{G_{2}}=\lambda \times \frac{P_{start}-P_{near}}{\left\|P_{start}-P_{near}\right\|}$$

where $\lambda \left(0\leq \lambda \leq 1\right)$ is an attractive gain factor. Considering convex polygonal obstacles $O=\left\{O_{1},O_{2},\ldots ,O_{m}\right\}$, the repulsive force exerted by obstacle $O_{i}$ on node q is:

(7) $$\vec{H}=\sum_{O_{i}\in O}\left\{\begin{array}{l} \xi \cdot \left(\frac{1}{d_{i}}-\frac{1}{d_{0}}\right)\cdot \frac{p-q_{i}}{d_{i}^{3}},d_{i}\leq d_{0}\\ 0,\;\;\;\;\;\;\;d_{i}>d_{0}\; \end{array}\right.$$

where ξ is the repulsive gain coefficient; $q_{i}$ is the point on the obstacle $O_{i}$ that is closest to the current node p (determined by calculating the shortest distance from p to each edge of the obstacle $O_{i}$); $d_{i}=\left\|p-q_{i}\right\|$ is the Euclidean distance from the node p to the nearest point $q_{i}$ on the obstacle; $d_{0}$ is the repulsion force influence range threshold, beyond which obstacles do not generate a repulsion force. The repulsion force direction is opposite to the direction from p to (i.e., away from the obstacle), and its magnitude decreases with $d_{i}$ increasing distance, ensuring that the repulsion force is significant at close distances. The new node generation strategy is then modified as shown in Figure 8 and Equation (8):

(8) $$P_{new}=P_{near}+s\cdot \vec{F}+\vec{G_{i}}$$

where $s^{\prime }$ is a dynamically adjusted step size (see Section 3.4). This mechanism provides target-oriented growth while preserving random exploration capability, significantly increasing the probability of tree connection.

### 3.4. Adaptive Step Size Based on Obstacle Density

To handle uneven obstacle distributions, an adaptive step size method based on the Sigmoid function [21] is proposed:

(9) $$s^{\prime }=s_{\max}\cdot \frac{1}{1+e^{-\alpha \cdot \left(d_{obs}-h\right)}}$$

where $s_{\max}$ is the maximum fixed step size, $d_{obs}$ is the distance from the node to the nearest obstacle, h is a safety threshold, and α is a scaling factor. When $d_{obs}\geq h$ (sparse obstacles), $s^{\prime }$ approaches $s_{\max}$ to enhance expansion efficiency. When $d_{obs}<h$ (dense obstacles), $s^{\prime }$ nonlinearly decreases towards a minimum value, mitigating collision risk from large steps and reducing path detours.

### 3.5. Obstacle Avoidance and Collision Detection Mechanism

Obstacle avoidance and collision detection are core technologies for ensuring the safe movement of intelligent agents in complex environments, directly affecting the reliability and practicality of path planning. This section details how the KDB-RRT* algorithm combines a multi-level collision detection framework with an active obstacle avoidance strategy to efficiently generate collision-free paths.

#### 3.5.1. Collision Detection Framework

The core objective of collision detection is to determine whether there is a spatial intersection (including contact) between the path segment and the obstacle boundary, providing strict verification of path safety. KDB-RRT* uses a precise geometric detection method based on polygon boundaries, balancing detection accuracy and computational efficiency. The key steps are as follows:

1. Obstacle Modeling: In the 2D planning space, all obstacles are modeled as a set of polygons $O=\left\{O_{1},O_{2},\ldots ,O_{k}\right\}$. Each obstacle $O_{i}$ is defined by an ordered sequence of vertices $\left\{v_{1},v_{2},\ldots ,v_{m}\right\}$ (vertices are arranged in a clockwise order to ensure polygon closure, with $v_{m+1}=v_{1}$).
2. Collision Criterion: For any path segment $L=P_{a}P_{b}$ (where $P_{a}$ and $P_{b}$ are consecutive nodes on the path), the necessary and sufficient condition for a collision with obstacle $O_{i}$ is that L intersects with any boundary edge of $O_{i}$ (including endpoint contact) or L lies entirely inside $O_{i}$. Mathematically, it can be formalized as:
  (10) $$Collision\left(L,O_{i}\right)=\left\{\begin{array}{l} true,\;\;if\exists j\in \left[1,m\right]\;s.t.\;L\cap v_{j}v_{j+1}\neq \emptyset \\ false,\;otherwise \end{array}\right.$$
  where $v_{j}v_{j+1}$ denotes the j boundary edge of obstacle $O_{i}$; the operator ∩ represents the intersection relationship of geometric line segments (including endpoint contact). If the judgment is true, the path segment L is invalid with a collision.
3. Detection Acceleration Technologies: To address the inefficiency of detection in large-scale obstacle scenarios, this study introduces two key optimizations:

Separating Axis Theorem (SAT) Pre-judgment: For polygonal obstacles, SAT is used to achieve fast collision judgment through projection analysis. Calculate the projection intervals of L and $O_{i}$ on multiple feature axes (including the normal vectors of each edge of $O_{i}$ and the normal vector of L). If there exists any axis where the projection intervals do not overlap, it can be directly determined that L and $O_{i}$ are collision-free without verifying all boundary edges. This method reduces the average time complexity of single obstacle detection from $O\left(m\right)$ (where m is the number of edges of the obstacle) to $O\left(1\right)$.
Spatial Index Filtering (R-tree): In the preprocessing phase, an R-tree spatial index structure for obstacle boundary edges is constructed, partitioning them into several sub-regions based on the spatial coordinates (bounding boxes) of the boundary edges. When detecting path segment L, the R-tree is first used to quickly retrieve the set of candidate boundary edges that are spatially intersecting or adjacent to L (with a distance less than a preset threshold). Only these candidate edges are subjected to precise intersection verification and inner point judgment using SAT.

#### 3.5.2. Active Obstacle Avoidance Mechanism

In addition to passive collision detection, KDB-RRT* introduces a three-level active obstacle avoidance strategy to dynamically adjust expansion behavior during path tree growth, achieving a “safety-efficiency” balance:

1. Potential Field-Guided Growth Control: As described in Section 3.3, the repulsive force component generated by the potential field directly guides new nodes away from obstacles in the node expansion direction (Equation (7)), proactively avoiding potential collision areas.
2. Adaptive Step-Size Control Based on Obstacle Density: As described in Section 3.4, the adaptive step size is dynamically adjusted according to the distance from the current node to the nearest obstacle. When approaching an obstacle, the step size is automatically reduced to lower the probability of path segments crossing obstacles due to excessively large step sizes and improve path quality in narrow areas.
3. Safety Buffer Expansion: Considering the agent’s own physical size and uncertainty factors such as motion control and positioning errors, the original obstacle boundaries are expanded to construct a safety buffer zone, ensuring that the planned path maintains a minimum safe distance $d_{safe\_\min}$ from the original obstacles. The minimum safe distance is defined as:
  (11) $$d_{safe\_\min}=R_{rob}+\delta_{s}$$
  where $R_{rob}$ denotes the radius of the agent (since the agent is modeled as a mass point in this study, $R_{rob}=0$), and $\delta_{s}$ is an additional safety margin to cope with uncertainties.

**Figure 9:**
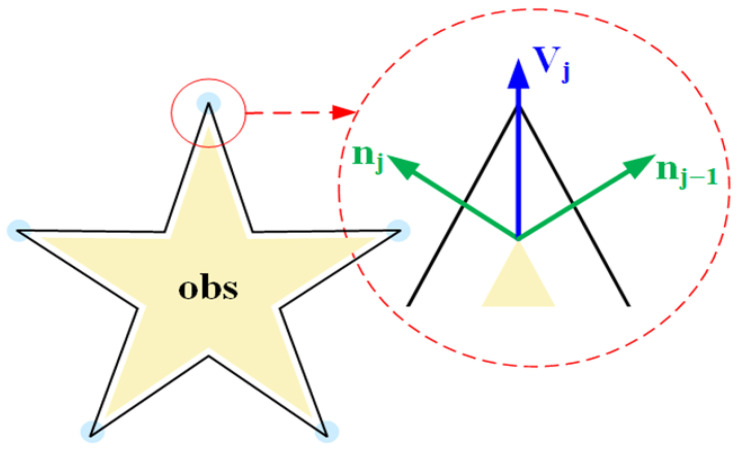
Schematic diagram of safety buffer zone.

For each vertex $v_{j}$ of each obstacle $O_{i}$, calculate the unit normal directions $n_{j-1}$ and $n_{j}$ of its adjacent edges ($v_{j-1}v_{j}$ and $v_{j}v_{j+1}$), and extrapolate a distance $d_{safe\_\min}$ to obtain a new vertex $v_{j}^{new}$. The new vertex $v_{j}^{new}$ is defined as shown in Equation (12). The schematic diagram of the safety buffer zone is shown in Figure 9, where the blue circles indicate some of the vertices of the polygon; the yellow pentagon represents the original obstacle (obs); and red dashed circles depict the safety buffer zone, with the enlarged circle at the vertex highlighting the expansion effect near sharp edges.

(12) $$v_{j}^{new}=v_{j}+d_{safe\_\min}\cdot \frac{n_{j-1}+n_{j}}{\left\|n_{j-1}+n_{j}\right\|}$$

Connecting all new vertices forms the new polygonal boundary $O_{i}^{buffered}$. Collision detection (Section 3.5.1) is entirely performed based on this expanded safety boundary $O_{i}^{buffered}$, thereby physically ensuring the safety of the agent’s movement and avoiding mechanical collisions caused by paths adhering too closely to the original obstacles.

### 3.6. Path Optimization

#### 3.6.1. Pruning

While the aforementioned optimization strategies significantly reduce redundant path points, some remain. Therefore, the Douglas–Peucker (DP) algorithm [22] is employed for path simplification.

The core idea of the DP algorithm is to recursively simplify a polyline into a polyline with fewer points, while retaining the main features of the original polyline as much as possible.

**Figure 10:**
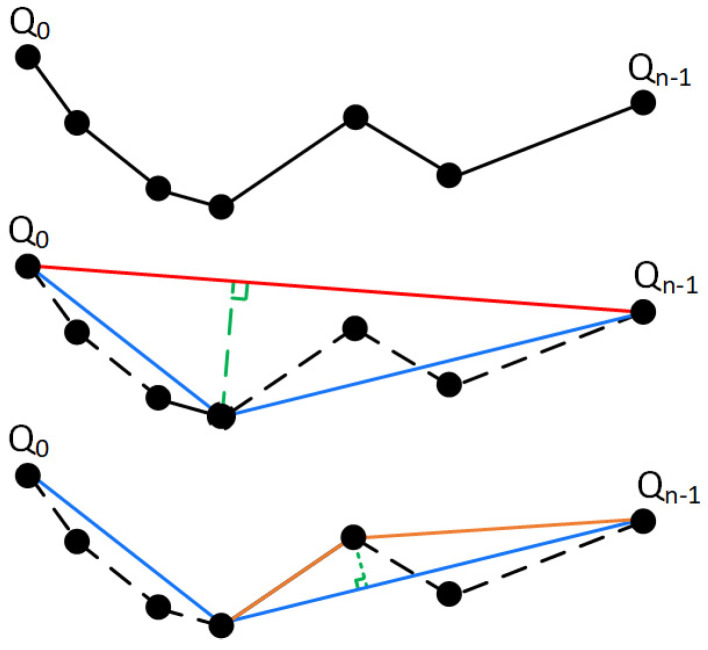
DP algorithm pruning.

Suppose a path point sequence ${\left\{Q_{i}\right\}}_{i=0}^{n}$, a tolerance threshold D, start point $Q_{0}$, and end point $Q_{n-1}$. The process of pruning using the DP algorithm is as follows, as shown in Figure 10.

In Figure 10, the solid red line represents the connection between the start point and end points, i.e., the line linking $Q_{0}Q_{n-1}$. The dashed green line indicates the perpendicular distance from an intermediate point to this line segment. The solid blue and orange lines depict the subpaths generated during recursive division (blue represents the first division, while orange denotes subsequent divisions).

1. $Q_{0}$ and last point $Q_{n-1}$ of the curve;
2. Calculate the perpendicular distance $d_{i}$ from each intermediate point $Q_{i}\left(1\leq i\leq n-2\right)$ to the line segment $Q_{0}Q_{n-1}$. Find the point $Q_{\max}$ with the maximum distance $d_{\max}$;
3. If $d_{\max}\leq D$, simplify the path to $\left\{Q_{0},Q_{n-1}\right\}$. If $d_{\max}>D$, split the path at $Q_{\max}$ into two sub-paths: $\left\{Q_{0},Q_{1},\ldots ,Q_{\max}\right\}$ and $\left\{Q_{\max},\ldots ,Q_{n-1}\right\}$;
4. Repeat steps 1–3 for all path curve segments until all $d_{\max}$ values are less than D. This completes the thinning process.

The geometric simplification process of the DP algorithm may lead to path intrusion into obstacle regions. Therefore, the following obstacle avoidance and safety guarantee mechanisms are introduced:

1. Injection of safety constraints: When calculating the maximum perpendicular distance $d_{\max}$, the minimum distance between the simplified path segment and obstacles is simultaneously verified. If a simplification operation results in the distance $d_{obs}$ from the path segment to the nearest obstacle falling below the safety threshold $d_{safe\_\min}$ (Equation (11)), the current simplification operation is aborted.
2. Spatial index acceleration: A pre-constructed R-tree spatial index is utilized to quickly retrieve candidate obstacle edges within the neighborhood of the path segment. Only the candidate set undergoes precise distance calculation, avoiding global traversal of all obstacles.

#### 3.6.2. Smoothing

**Figure 11:**
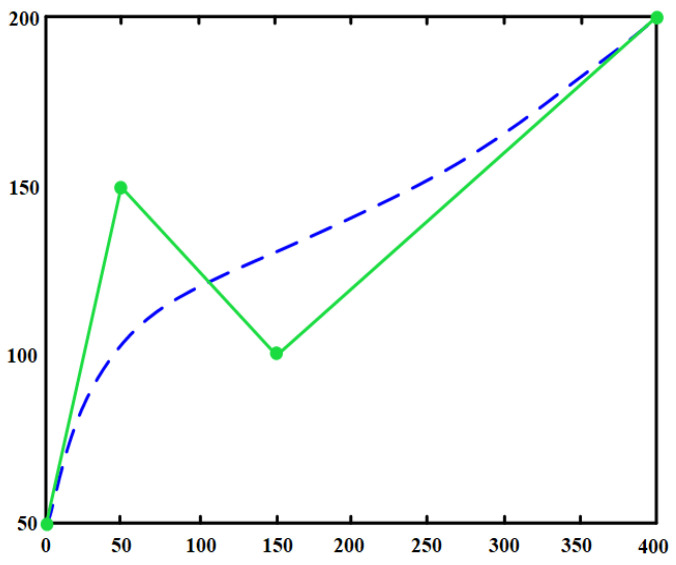
B-spline curve.

To reduce the curvature of the path and make it smoother, this paper uses cubic B-spline curves [23] to smooth the pruned path. A cubic B-spline curve is inserted between path segments, connecting discrete points into a smooth curve. By adjusting the positions and weights of control points, the path shape can be further refined. Given the path point sequence ${\left\{H_{i}\right\}}_{i=0}^{n}$, construct the parametric curve:

(13) $$C\left(u\right)=\left[P_{0},P_{1},\ldots ,P_{n}\right]\left[\begin{array}{l} N_{0,3}\left(u\right)\\ N_{1,3}\left(u\right)\\ N_{2,3}\left(u\right)\\ N_{3,3}\left(u\right) \end{array}\right]=\sum_{j=0}^{n}P_{j}\cdot N_{j,3}\left(u\right)$$

where $N_{j,3}$ are the cubic B-spline basis functions, and the control points $\left\{P_{j}\right\}\left(j=0,1,\ldots ,m\right)$ are fitted to the pruned path using least squares. A curvature constraint optimization model is introduced:

(14) $$\underset{p}{\min}\sum_{i=0}^{n}{\left\|P\left(u_{i}\right)-H_{i}\right\|}^{2}+\gamma \int_{0}^{1}\kappa^{2}\left(u\right)du$$

where $\kappa \left(u\right)=\frac{\left\|C^{\prime }\left(u\right)\times C^{\prime \prime }\left(u\right)\right\|}{{\left\|C^{\prime \prime }\left(u\right)\right\|}^{3}}$ is the curvature at $P_{i}$, and γ is a smoothness weighting coefficient. Four control points from the random tree are used within the cubic B-spline function to generate control points. Connecting these points in sequence produces the smooth path shown in Figure 11, where the green polyline represents the pruned discrete path (before smoothing), and the blue curve denotes the cubic B-spline-generated smooth path, effectively modifying local curvature while preserving the overall path topology.

### 3.7. Algorithm Implementation

**Figure 12:**
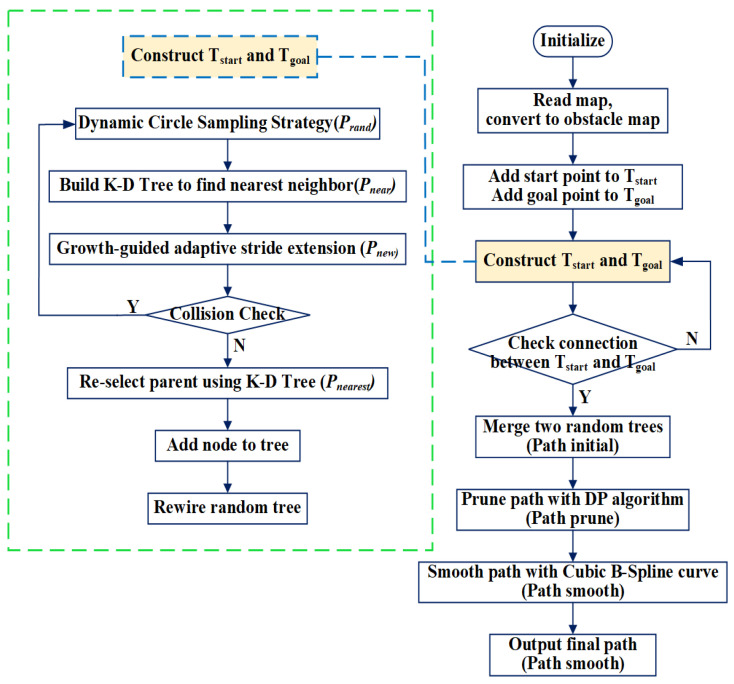
KDB-RRT* algorithm flowchart.

**Figure 13:**
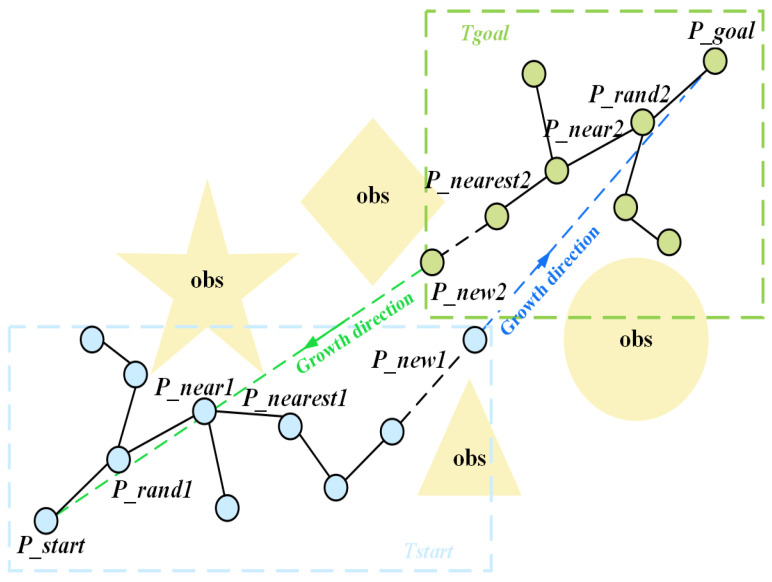
Basic principle diagram of KDB-RRT*.

Based on the aforementioned improvements, a KD-tree-based goal-biased dual RRT* algorithm (KDB-RRT*) is proposed to ensure rapid planning of collision-free, near-optimal paths for agents. Referring to the KDB-RRT* flowchart (Figure 12) and schematic (Figure 13), the key steps of the KDB-RRT* algorithm are outlined below:

Where in Figure 12, the yellow rectangular box represents the growth process of $T_{start}$ and $T_{goal}$, as detailed in the procedure outlined within the green rectangular box on the left; and in Figure 13, the yellow polygons represent obstacles, the blue and green rectangular boxes denote the construction of $T_{start}$ and $T_{goal}$, the blue and green dashed lines indicate the growth directions of $T_{start}$ and $T_{goal}$, the blue and green circles represent the nodes in $T_{start}$ and $T_{goal}$.

1. Initialize: Map information, parameters, start ($P_{start}$), and goal ($P_{goal}$) are loaded. Initialize trees: $T_{start}$ with $P_{start}$ and $T_{goal}$ with $P_{goal}$.
2. Sample: For the current tree (e.g., $T_{start}$), generate sampling points within the dynamic circular sampling region Ω with center $P_{i}$ and radius $R\left(d\right)$ (Section 3.2, Equations (3) and (4)).
3. Find Nearest Neighbor ($P_{\mathrm{near}}$): Using the KD-Tree (Section 3.1) built for the current tree, perform a nearest neighbor search to find $P_{\mathrm{near}}$ closest to $P_{rand}$.
4. Steer: Generate a new node $P_{\mathrm{new}}$ using the growth guidance model (Section 3.3 and Section 3.4, Equations (6)–(8)) with the adaptive step size (Equation (9)).
5. Collision Check: Verify if $P_{\mathrm{new}}$ is collision-free and if the path segment connecting $P_{\mathrm{near}}$ to $P_{\mathrm{new}}$ is collision-free (Section 3.5).
6. Re-select Parent: If collision-free, use the KD-Tree to find the set of potential parent nodes $N_{parent}$ near $P_{\mathrm{new}}$. Select the node $P_{\mathrm{nearest}}$ that provides the minimum cost path to $P_{\mathrm{new}}$ and set it as the parent of $P_{\mathrm{new}}$.
7. Add Node: Add the valid $P_{\mathrm{nearest}}$ to the current tree.
8. Rewire the tree: For nodes $P_{\mathrm{near}}$ in $N_{parent}$, check if connecting them to $P_{\mathrm{new}}$ provides a lower-cost path. If so, rewire their parent to $P_{\mathrm{new}}$.
9. Check Connection: Check if the latest added node in $T_{start}$ and the latest added node in $T_{goal}$ can connect (distance between them less than or equal to connection threshold or the latest added node in $T_{start}$ is near a node in $T_{goal}$ or the latest added node in $T_{goal}$ is near a node in $T_{start}$) via a collision-free path.
10. Tree Connection: If connected, merge the trees $T_{start}$ and $T_{goal}$ at the connection point to form the initial path Path_initial. If not connected, return to Step 2 and continue constructing two trees.
11. Path Pruning: Apply the DP algorithm (Section 3.6) to Path_initial using threshold D to remove redundant points, yielding the simplified path Path_pruned.
12. Path Smoothing: Apply cubic B-spline fitting (Section 3.6, Equations (13) and (14)) to Path_pruned generate the final smooth path Path_smooth.
13. Output: Return the optimized path Path_smooth.

### 3.8. Computational Complexity Analysis of KDB-RRT*

The time complexity of KDB-RRT* is systematically compared with classical RRT* in Table 1. The analysis considers N as the number of nodes in the search tree(s), M as the number of obstacle edges, k as the number of iterations, and L as the number of path nodes in the final solution.

## 4. Algorithm Testing and Simulation

### 4.1. Simulation Experiment Design

This study employs MATLAB R2022a and the ROS Noetic (Robot Operating System) robotic experimental platform to validate the performance of the proposed algorithm across multiple dimensions, including feasibility, superiority, and reliability. The laptop specifications are: Windows 11 operating system, 13th Gen Intel^®®^ Core^TM^ i7-13700H 2.40 GHz processor, 16.0 GB RAM (Intel, Santa Clara, CA, USA); utilizing a virtual machine with Ubuntu 20.04.6.

**Figure 14:**
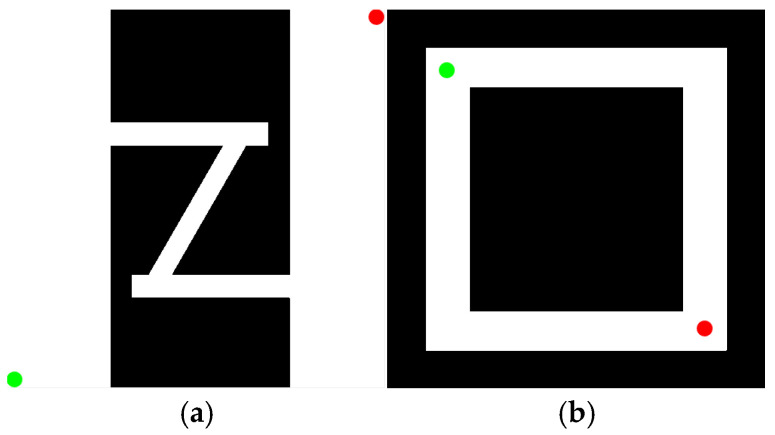
Simulation Environment 1. (**a**) “Z-shaped” map; (**b**) “loop-shaped” map.

To verify the feasibility of the KDB-RRT* algorithm, two simulation environments were established, as shown in Figure 14. The map dimensions for both environments were set to 500 × 500. Black regions represent obstacles, while white regions denote free space.

“Z-Shaped” Map (Figure 14a): Start point (green circle) at (1, 500), goal point (red circle) at (500, 1).

“Loop-Shaped” Map (Figure 14b): Start point (green circle) at (20, 20), goal point (red circle) at (480, 480).

**Figure 15:**
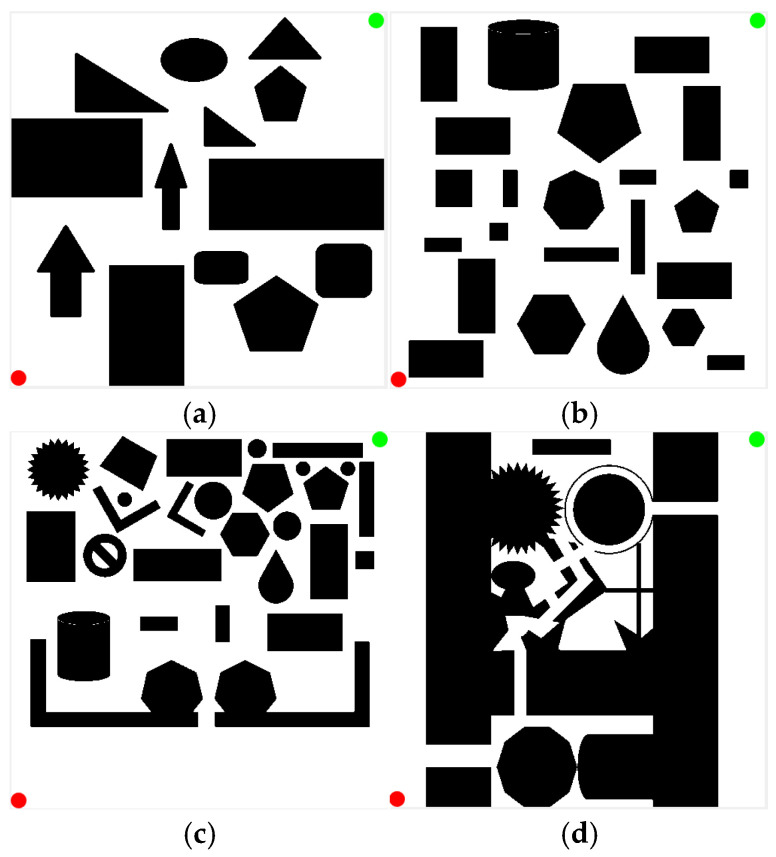
Simulation Environment 2. (**a**) Map1; (**b**) Map2; (**c**) Map3; (**d**) Map4.

To demonstrate the superiority of the KDB-RRT* algorithm, four simulation environments were established, as shown in Figure 15. The map dimensions were set to 500 × 500, with the start point (red circle) at (1, 500) and the goal point (green circle) at (500, 1) for all environments.

### 4.2. Feasibility of KDB-RRT*

**Figure 16:**
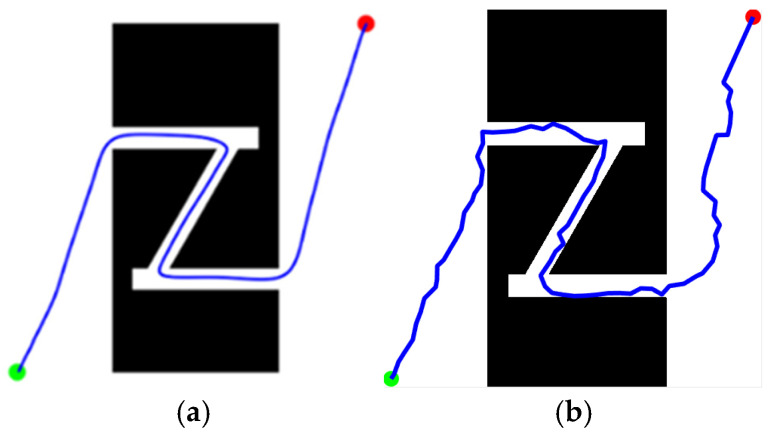
Experimental Results for the “Z-shaped” Environment. (**a**) KDB-RRT*; (**b**) Optimized RRT*.

**Figure 17:**
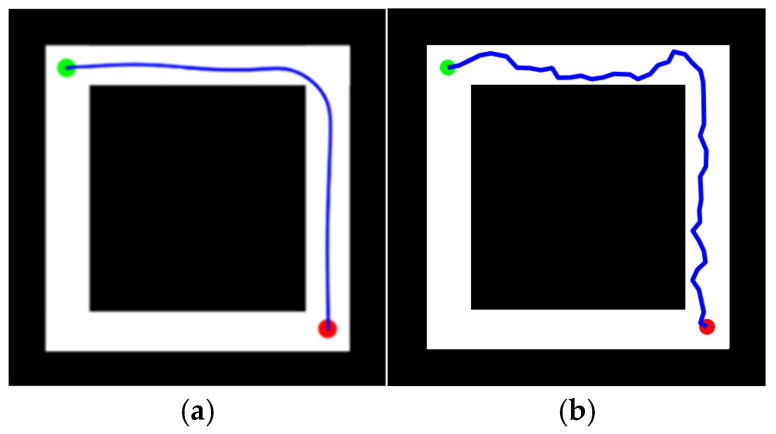
Experimental Results for the “Loop-shaped” Environment. (**a**) KDB-RRT*; (**b**) Optimized RRT*.

Path planning tasks were executed on the “Z-Shaped” and “Loop-Shaped” maps (Section 4.1, Figure 14) using both the proposed KDB-RRT* algorithm (incorporating the KD-Tree) and the Optimized RRT* algorithm. Parameters were set as: goal connection threshold = 20, safety threshold = 1, maximum step size = 30, and maximum iterations = 5000. Each experiment was repeated 50 times, and average values were computed. The simulation results (Figure 16 and Figure 17) demonstrate that the KDB-RRT* algorithm exhibits robust path planning capabilities in both typical complex environments. Core metrics are summarized in Table 2 and Table 3.

Z-Shaped Map (Figure 16): KDB-RRT* (Figure 16a): The planned path navigates the narrow “Z” corridor with minimal adjustments primarily at key turning points. This indicates the algorithm’s ability to rapidly identify geometric features of narrow passages, reducing redundant exploration. The path maintains precise clearance from obstacles without local oscillations, demonstrating an effective balance between smoothness and safety in unstructured narrow channels. Optimized RRT* (Figure 16b): The path exhibits frequent zig-zag turns within the corridor, resulting from redundant node expansion due to random sampling. Some segments show extreme proximity to obstacles (especially at entrances), indicating that the lack of dynamic biasing leads to conservative but suboptimal paths. The average planning time of KDB-RRT* (4.10 s) was 70.2% shorter than that of the Optimized RRT* (13.77 s). The average path length of KDB-RRT* (1258.25 cm) was 4.8% shorter than that of the Optimized RRT* (1321.92 cm). The average number of path nodes of KDB-RRT* decreased by 67.5%, and the node utilization rate increased by 16.2%. The average distance to obstacles (5.23 cm) of KDB-RRT* was 2.36 cm greater than that of Optimized RRT* (2.17 cm). The minimum distance to obstacles was 4.69 cm, which meets the safety threshold, representing an increase of 3.33 cm compared to the Optimized RRT*.

Loop-Shaped Map (Figure 17): KDB-RRT* (Figure 17a): The path successfully traverses the nested obstacle gaps within the loop environment, forming a globally optimized trajectory from the start (green point) to the goal (red point). Turns exhibit uniform curvature without sharp corners, making it suitable for agent motion control. Optimized RRT * (Figure 17b): The path shows multiple detours near the middle obstacle layer, reflecting entrapment in local minima requiring backtracking to escape invalid regions. The total path length is significantly longer than KDB-RRT*, incurring extra cost, particularly when navigating the outer loop structure. The average planning time of KDB-RRT* (0.85 s) was only 28.0% of that of the Optimized RRT* (3.04 s). The average path length of KDB-RRT* (646.47 cm) was 10.4% shorter than that of the Optimized RRT* (721.68 cm). The average number of path nodes of KDB-RRT* decreased by 69.6%, and the node utilization rate increased by 13.5%. The average distance to obstacles (4.36 cm) of KDB-RRT* exceeded that of Optimized RRT* (1.89 cm) by 2.47 cm, demonstrating superior obstacle-awareness in the nested loop structure. The minimum distance of 3.74 cm further confirmed stable collision avoidance, contrasting with the marginal 1.27 cm of Optimized RRT*.

### 4.3. Superiority of KDB-RRT*

#### 4.3.1. Superiority of KD-Tree Integration

Path planning tasks were executed on Map 1 (Section 4.1, Figure 15a) using the Optimized RRT* algorithm and the KDB-RRT* algorithm incorporating the KD-Tree. Parameters: goal threshold = 20, safety threshold = 1, initial step size = 30, max iterations = 5000. Each experiment was repeated 50 times. Results are shown in Figure 17.

**Figure 18:**
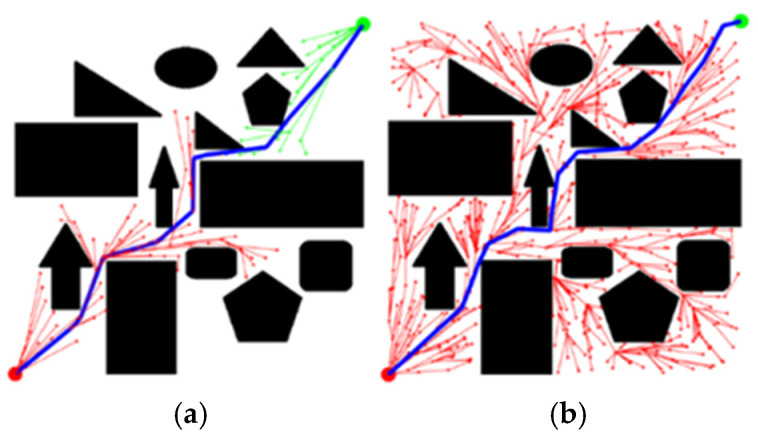
Comparative experimental results of path superiority with the introduction of the KD-tree. (**a**) KDB-RRT*; (**b**) optimized RRT*.

Figure 18 demonstrates that the KDB-RRT* algorithm with KD-Tree optimization (Figure 18b) exhibits significantly fewer node exploration attempts (number of lines other than the blue path) compared to the optimized RRT* (Figure 18a), where the blue lines represent paths, the lines outside the blue lines represent the search process, and the two colors of lines in Figure 18a represent two tree searches. The KDB-RRT* path also displays higher smoothness and directional continuity. The fewer red and green lines in Figure 18b correspond intuitively to the greater number of red lines in Figure 18a, as well as the sparsification of path inflection points in Figure 18b compared to the dense, sawtooth-like fluctuations in the path of Figure 18a. This optimization is achieved through the KD tree’s dynamic hierarchical indexing mechanism in high-dimensional space. By dynamically adjusting the connection strategy between neighboring nodes, it prioritizes the directionally optimal connection from the parent node to the target point, thereby reducing path complexity while improving search efficiency. This makes the path more suitable for the stringent requirements of real-time performance, low energy consumption, and stable movement during the agent’s operation. The comparison of core metrics before and after optimization is shown in Table 4. Comparative data shows: Average planning time was reduced from 12.95 s (Optimized RRT*) to 1.13 s (KDB-RRT*), representing a 91.3% improvement. Average path length was reduced from 790.79 cm to 747.06 cm, a reduction of 43.73 cm (5.5%). Average iterations were reduced by 91.0%. Average node utilization rate exhibited a 39.39% increase. Additionally, KDB-RRT* achieves a 2.56 cm average distance to obstacles, 57% higher than Optimized RRT* (1.63 cm), and a 1.38 cm minimum distance, 11% higher than Optimized RRT* (1.24 cm). The KD-Tree’s efficient neighbor search enables the algorithm to implicitly bias sampling toward “safe-optimal” regions, avoiding the marginal safety (1.24 cm, only 0.24 cm above the threshold) observed in Optimized RRT*.

#### 4.3.2. Superiority of DP Pruning

**Figure 19:**
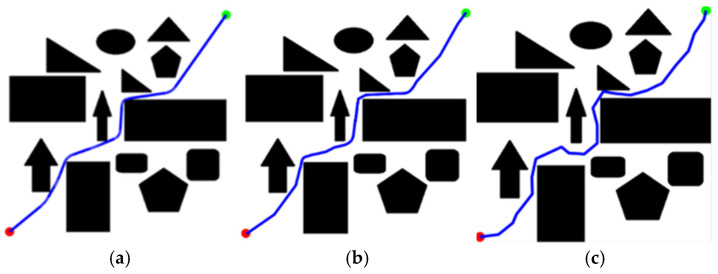
Comparative experimental results on the superiority of path pruning with the DP algorithm. (**a**) DP algorithm pruning; (**b**) triangular pruning; (**c**) before pruning.

The initial path planned by the KDB-RRT* algorithm (incorporating the KD-Tree) on Map 1 (Section 4.1, Figure 15a) was processed using the DP algorithm for pruning. The pruned path using DP, the path pruned using triangular pruning, and the original unpruned path are compared in Figure 19.

Figure 19 shows that in the complex environment with dense black geometric obstacles, both DP pruning (Figure 19a) and triangular pruning (Figure 19b) effectively remove redundant inflection points present in the unpruned path (Figure 19c), significantly improving overall path smoothness and continuity. However, compared to triangular pruning, the DP-pruned path demonstrates greater advantages: in terms of path length: 742.23 cm, 1.97% shorter than triangular pruning and 2.7% shorter than the unpruned path; in terms of the number of inflection points: 5, a reduction of 54.5% compared to triangular pruning and 72.2% compared to the unpruned path; in terms of average processing time: 0.67 s, 39.3% faster than triangular pruning. DP pruning achieves a 2.48 cm average distance to obstacles (6.8% lower than triangular pruning’s 2.65 cm, but 4.6% higher than the unpruned path’s 2.37 cm) and a 1.65 cm minimum distance (3.5% lower than triangular pruning’s 1.71 cm, but 1.8% higher than the unpruned path’s 1.62 cm). This indicates DP pruning reduces unnecessary safety margins (compared to triangular pruning) while maintaining a stable buffer above the 1 cm threshold.

These results stem from DP’s safety-constrained simplification logic: during pruning, the algorithm checks the minimum distance of each path segment to obstacles; if the distance approaches the safety threshold, pruning is terminated for that segment. This ensures the simplified path retains geometric optimality without compromising collision resistance. Thus, DP pruning simultaneously reduces energy consumption (via fewer turns) and enhances navigation stability, while preserving a robust safety margin. Core path metrics before and after optimization are compared in Table 5.

#### 4.3.3. Simulation Experiment Based on KDB-RRT*

**Figure 20:**
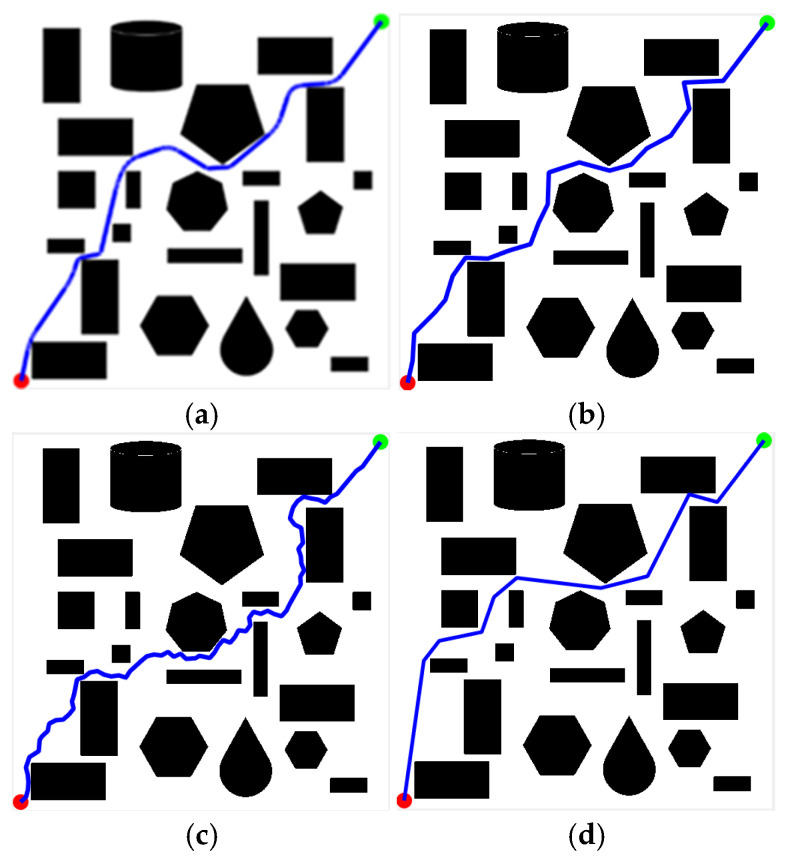
Comparative experimental results of different algorithms for Map 2. (**a**) KDB-RRT*; (**b**) Optimized RRT*; (**c**) RRT*-Connect; (**d**) Informed-RRT*.

**Figure 21:**
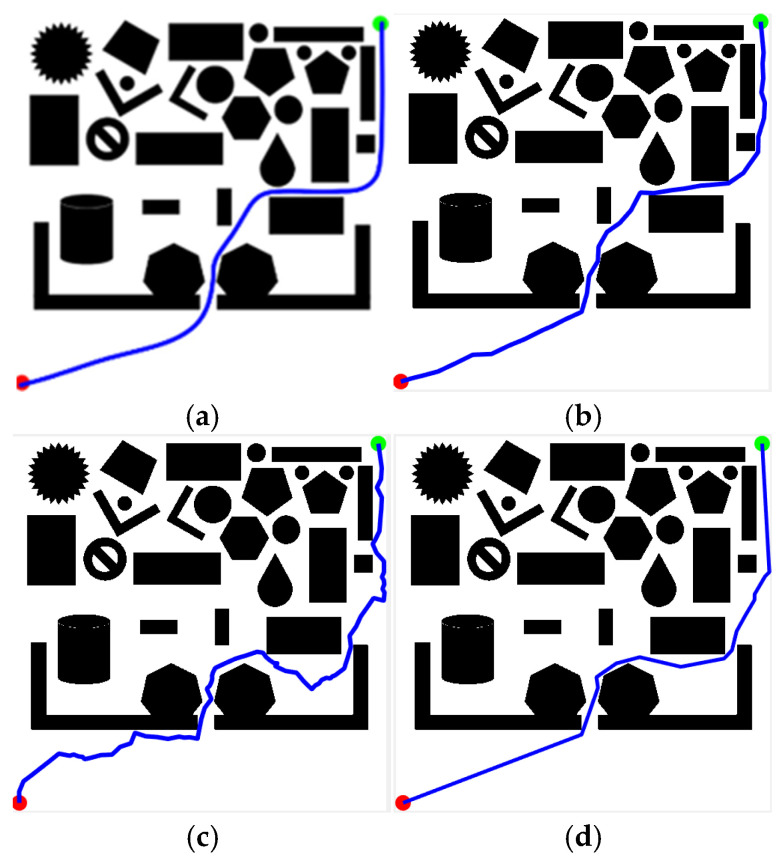
Comparative experimental results of different algorithms for Map 3. (**a**) KDB-RRT*; (**b**) Optimized RRT*; (**c**) RRT*-Connect; (**d**) Informed-RRT*.

**Figure 22:**
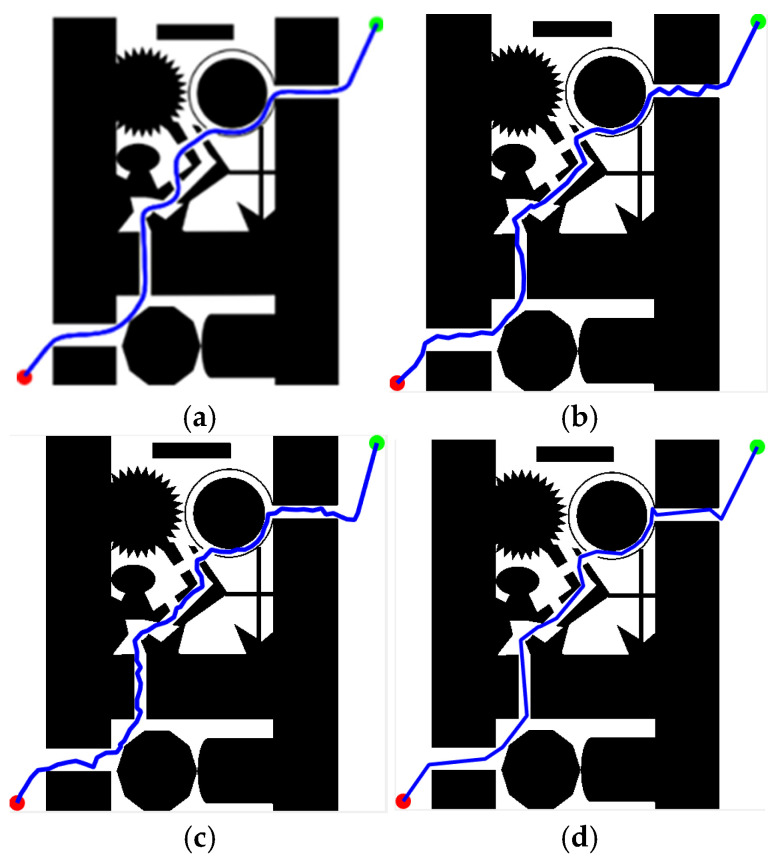
Comparative experimental results of different algorithms for Map 4. (**a**) KDB-RRT*; (**b**) Optimized RRT*; (**c**) RRT*-Connect; (**d**) Informed-RRT*.

To validate the superiority of the proposed KDB-RRT* algorithm, comparative experiments were conducted across diverse simulation environments (Section 4.1, Figure 15b–d) against Optimized RRT*, RRT*-Connect, and Informed-RRT*. Performance was analyzed based on four key metrics: average planning time, average path length, average number of inflection points, and number of planning failures. Parameters: goal threshold = 20, safety threshold = 1, max step size = 30, max iterations = 5000. Each experiment was repeated 50 times. Results for different maps are shown in Figure 20, Figure 21 and Figure 22.

Figure 20 shows the path planning results of the four algorithms in Map2 (Section 4.1, Figure 15b) under consistent basic parameters. From the experimental results, it can be seen that KDB-RRT* (Figure 20a) generates a continuous, smooth curve navigating dense obstacle gaps, with necessary turns only at critical bottlenecks. Optimized RRT* (Figure 20b) exhibits frequent backtracking in narrow passages. RRT*-Connect (Figure 20c) produces paths with numerous sharp turns. Informed-RRT* (Figure 20d) reduces curvature changes but still features large-angle turns.

Core metrics are summarized in Table 6. All algorithms achieved a 100% planning success rate, validating the navigability of Map 2’s complex obstacle layout. For KDB-RRT*, its average planning time of 1.11 s represents a 95.8% (vs. Optimized RRT*), 95.4% (vs. RRT*-Connect), and 77.2% (vs. Informed-RRT*) reduction, underscoring superior computational efficiency. In path quality, the average path length of 774.89 cm is 1.1% shorter than Optimized RRT*, 7.27% shorter than RRT*-Connect, and 0.67% shorter than Informed-RRT*, while the average number of inflection points (9) decreases by 65.4%, 78.0%, and 10.0% compared to the three algorithms, respectively—indicating smoother trajectory generation. Critical to safety, KDB-RRT* exhibits a 2.05 cm average distance to obstacles, which is 19.2% and 21.9% higher than Optimized RRT* (1.72 cm) and RRT*-Connect (1.68 cm), respectively, demonstrating a more robust global safety margin. Its 1.39 cm minimum distance to obstacles exceeds Optimized RRT* (1.33 cm) by 0.06 cm and RRT*-Connect (1.31 cm) by 0.08 cm, while remaining only 0.02 cm lower than Informed-RRT* (1.41 cm). This near-parity with Informed-RRT* (a safety-focused algorithm) highlights KDB-RRT*’s ability to balance safety and optimality: via the dynamic gravitational field (Section 3.3), it steers paths to maintain just-sufficient clearance (avoiding excessive detours) while ensuring compliance with the 1 cm safety threshold. Thus, KDB-RRT* simultaneously achieves faster planning, shorter paths, and competitive obstacle-aware safety—critical for real-time agent navigation in cluttered environments.

Map3 (Section 4.1, Figure 15c) is more complex than Map2. Figure 21 shows the path planning results of the four algorithms in Map3 under consistent basic parameters. From the experimental results, it can be seen that KDB-RRT* generates superior paths characterized by continuous smooth curves, adjusting direction only at critical narrow passages. Optimized RRT* paths show zig-zag fluctuations upon local magnification. RRT*-Connect paths exhibit numerous high-curvature turns. Informed-RRT* reduces turn count, but turn angles remain large.

Core metrics are summarized in Table 7. As shown in Table 7, KDB-RRT* demonstrates significant performance improvements: the average planning time of KDB-RRT* (1.17 s) is reduced by 97.5% compared to Optimized RRT*, by 96.9% compared to RRT*-Connect, and by 85.8% compared to Informed-RRT*—this is due to the nearest neighbor search of the KD tree (Section 3.1) and the dynamic circular sampling strategy with target bias (Section 3.2), which minimizes redundant node exploration. The average path length (792.48 cm) is 8.5% shorter than Optimized RRT*, 6.16% shorter than RRT*-Connect, and 2.45% shorter than Informed-RRT*, with only 4 turns—reductions of 84%, 90.5%, and 60%, respectively. This ensures smooth motion control, which is critical for real-world agent navigation. KDB-RRT* exhibits a 4.12 cm average distance to obstacles—a 2.14 cm (108%) and 2.27 cm (114%) increase over Optimized RRT* (1.98 cm) and RRT*-Connect (1.85 cm), respectively. Its 3.69 cm minimum distance to obstacles maintains a 2.69 cm margin above the 1 cm safety threshold, far outperforming the marginal safety of Optimized RRT* (0.35 cm above threshold) and RRT*-Connect (0.32 cm above threshold). While slightly lower than Informed-RRT* (4.37 cm average, 3.82 cm minimum), this near-parity confirms KDB-RRT* balances safety with efficiency: the adaptive step size mechanism (Section 3.4) compresses redundant clearance in constrained spaces without compromising collision resistance, avoiding the excessive detours inherent to purely safety-focused algorithms.

Notably, KDB-RRT* and Informed-RRT* achieve a 100% success rate, whereas Optimized RRT* (68%) and RRT*-Connect (72%) suffer frequent failures. This stark contrast highlights KDB-RRT*’s ability to avoid entrapment in local minima, enabled by the potential field’s goal-biased sampling (Section 3.3). By dynamically weighting repulsive forces from obstacles and attractive forces toward the goal, the algorithm prioritizes feasible paths through cluttered regions, even in complex topologies.

Map4 (Section 4.1, Figure 15d) is the most complex of the three maps. Figure 22 shows the path planning results of the four algorithms in Map4 under consistent basic parameters. Core metrics are summarized in Table 8. From the experimental results, it can be seen that KDB-RRT*, KDB-RRT* achieves a 100% planning success rate, significantly outperforming Optimized RRT* (28%), RRT*-Connect (36%), and even Informed-RRT* (98%). This robustness stems from the bidirectional growth model based on potential fields (Section 3.3): by leveraging the repulsive force of obstacles and the attractive force of the goal, the algorithm prioritizes feasible paths in cluttered areas, avoiding getting stuck in local optima—a critical advantage in the nested obstacle topology of Map 4. The average planning time of KDB-RRT* (1.96 s) is reduced by 97.9% compared to Optimized RRT*, by 95.9% compared to RRT*-Connect, and by 77.2% compared to Informed-RRT* by 77.2%—this is due to the KD-tree index-based nearest neighbor query optimization (Section 3.1), which minimizes redundant node exploration; and the target-biased dynamic circle sampling strategy (Section 3.2), which reduces the algorithm’s randomness. The average path length (791.88 cm) is 8.6% shorter than optimized RRT*, 21.4% shorter than RRT*-Connect, and 2.15% shorter than Informed-RRT*, with only 9 turns—reductions of 62.5%, 84.7%, and 40%, respectively. This smoothness is critical for motion control, stemming from pruning in the dynamic programming algorithm (Section 3.6), which removes redundant nodes while maintaining safety constraints. The average distance between KDB-RRT* and obstacles is 3.05 cm—43.2% higher than Optimized RRT* (2.13 cm) and 54.8% higher than RRT*-Connect (1.97 cm). Its minimum obstacle distance of 2.42 cm provides a safety margin of 1.42 cm, far exceeding the marginal safety levels of Optimized RRT* (0.57 cm) and RRT*-Connect (0.38 cm). Although Informed-RRT* has an average obstacle distance of 2.68 cm and a minimum distance of 2.04 cm, KDB-RRT* demonstrates a more balanced trade-off: The adaptive step size mechanism (Section 3.4) compresses redundant gaps in narrow passages without sacrificing collision resistance, avoiding the excessive detouring issues inherent in Informed-RRT*’s purely heuristic-driven sampling.

In summary, across diverse complex environments—including Z-shaped corridors, loop-shaped nested obstacles, and multi-obstacle cluttered maps—the proposed KDB-RRT* consistently outperforms state-of-the-art algorithms (Optimized RRT*, RRT*-Connect, and Informed-RRT*) in comprehensive performance metrics. In terms of planning efficiency, KDB-RRT* achieves a 70.2–97.9% reduction in average planning time (Table 2, Table 3, Table 4, Table 5, Table 6, Table 7 and Table 8), indicating that it can rapidly respond to path planning requests even in highly constrained scenarios. For path quality, KDB-RRT* generates 4.8–21.4% shorter paths with 40–90.5% fewer inflection points (Table 2, Table 3, Table 4, Table 5, Table 6, Table 7 and Table 8) compared to benchmark algorithms. This improvement stems from DP algorithm pruning (removing redundant nodes) and cubic B-spline smoothing, ensuring curvature-continuous trajectories suitable for stable agent motion control. Critically, in safety performance, KDB-RRT* maintains robust obstacle clearance: its average distance to obstacles and minimum distance to obstacles all exceed the 1 cm safety threshold (Table 2, Table 3, Table 4, Table 5, Table 6, Table 7 and Table 8). While its minimum distance is slightly lower than Informed-RRT* in some cases (e.g., 1.39 cm vs. 1.41 cm in Map 2), it achieves a superior balance between safety and optimality—avoiding excessive detours via gravitational field-guided repulsion and adaptive step size adjustment. Moreover, KDB-RRT* demonstrates 100% planning success rates across all environments, outperforming Optimized RRT* and RRT*-Connect in high-complexity maps (Table 7 and Table 8). These results collectively validate that KDB-RRT* integrates efficiency, optimality, and safety, making it a robust solution for agent path planning in unstructured and cluttered real-world scenarios.

### 4.4. Agent Path Planning Experiments Based on KDB-RRT*

**Figure 23:**
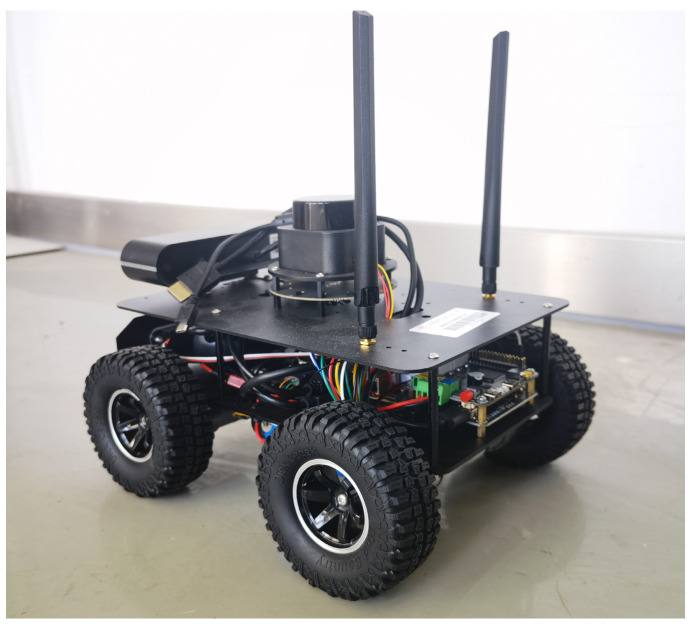
XTARK TARKBOT Series ROS Robot.

To validate the feasibility and reliability of the KDB-RRT* algorithm in practical agent applications, this study utilized the TARKBOT series ROS robot (Figure 23) as the experimental platform. The Simultaneous Localization and Mapping (SLAM) tools provided by ROS were used for environmental map modeling, and the Adaptive Monte Carlo Localization (AMCL) algorithm enabled real-time agent localization. Two typical static scenarios were designed to test the algorithm’s path planning capability under different obstacle distributions.

**Figure 24:**
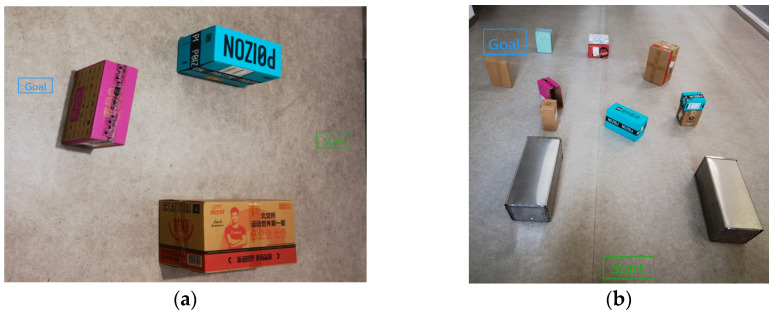
Experimental scenario setup for UGV path planning. (**a**) Scenario 1; (**b**) Scenario 2.

Scenario 1 (Figure 24a): A 4 m × 2 m area with 3 fixed obstacles arranged dispersedly, forming narrow passages. Start point (green) at the UGV’s initial position/origin (0,0), goal point (blue). The UGV’s right direction is the positive X-axis; the front direction is the positive Y-axis.

Scenario 2 (Figure 24b): A 10 m × 8 m area with 10 obstacles forming dense clusters and a “C-shaped” bend, simulating a complex indoor environment. Start and goal definitions same as Scenario 1.

To avoid experimental randomness, each scenario was tested 20 times, and average values were computed.

**Figure 25:**
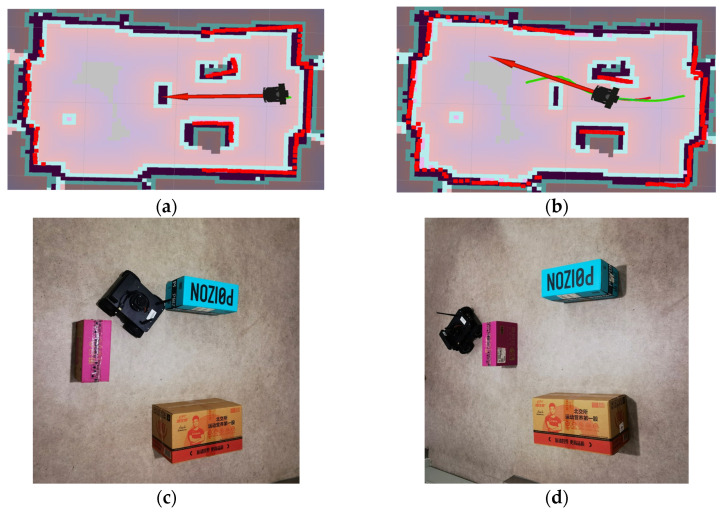
Path planning for UGV in scenario 1. (**a**) Initial diagram; (**b**) planning diagram; (**c**) process diagram; (**d**) result diagram.

In Scenario 1 (Figure 24a), the UGV initiates path planning based on the SLAM map depicted in Figure 25a, with the resultant path shown in Figure 25b, and the red arrows denote the travel direction of the Unmanned Ground Vehicle (UGV), and the green curve represents the planned path. Departing from the start point, the UGV effectively navigates obstacles during motion (Figure 25c), ultimately reaching the goal along a smooth trajectory (Figure 25d).

**Figure 26:**
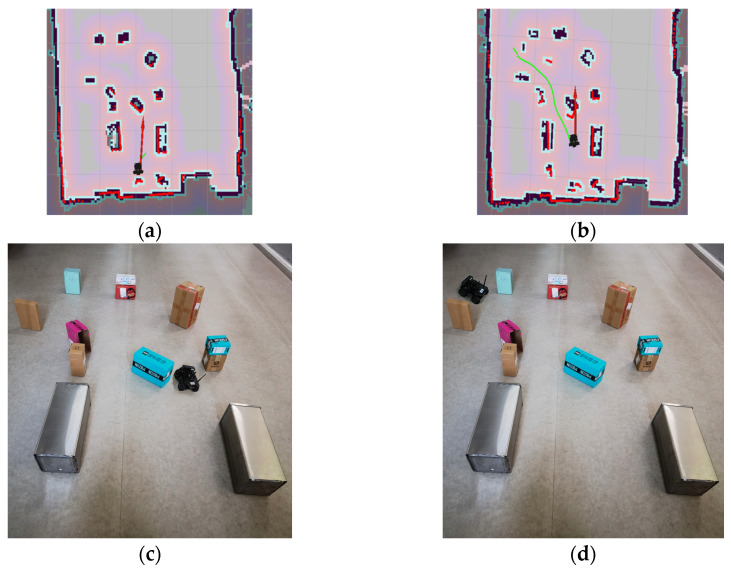
Path planning for UGV in scenario 2. (**a**) Initial diagram; (**b**) Planning diagram; (**c**) Process diagram; (**d**) Result diagram.

For Scenario 2 (Figure 24b), which requires traversal through dense obstacles including a “C-shaped” bend, planning commences using the SLAM map in Figure 26a. The generated path (Figure 26b) exhibits continuous curvature, enabling successful obstacle avoidance during movement (Figure 26c) and arrival at the goal via a smooth path (Figure 26d).

These results demonstrate consistent generation of near-optimal, collision-free paths with continuous curvature across both scenarios. Table 9 summarizes performance metrics from 20 experimental trials per scenario, including two critical distance-based indicators (unit: dm) to quantify obstacle-aware safety:

In Scenario 1 (sparse obstacle distribution), collision-free navigation was achieved with an average path length of 3.23 m, average planning time of 0.78 s, and average UGV travel time of 1.46 s. The average distance to obstacles reached 3.5 dm, with a minimum distance of 2.8 dm—both values far exceeding the predefined safety threshold (1 dm), indicating ample safety margins in less constrained environments.

For Scenario 2 (dense obstacles with a “C-shaped” corridor), collision-free operation was maintained with an average path length of 6.72 m, an average planning time of 1.64 s, and an average travel time of 3.08 s. Here, the average distance to obstacles decreased to 2.7 dm (27 cm) and the minimum distance to 1.3 dm (13 cm), yet both remained well above the safety threshold. This adaptive reduction in average distance reflects the algorithm’s ability to balance path optimality and safety via dynamic repulsion in the potential field model (Section 3.3), which compresses redundant clearance in constrained spaces without compromising collision-free constraints.

Collectively, these results confirm that the proposed KDB-RRT* algorithm generates near-optimal, collision-free paths across environments with varying obstacle densities. The consistent superiority of distance metrics over the safety threshold validates its robust obstacle-aware planning capability, underscoring feasibility for diverse agent path-planning scenarios.

## 5. Conclusions

Path planning stands as a core technology for intelligent agent navigation, directly determining the efficiency and safety of task execution in complex environments. Although RRT*-based algorithms possess probabilistic completeness, they typically suffer from high computational complexity, excessive path redundancy, and insufficient smoothness, limiting their practical application in unstructured scenarios. To address these limitations, this study proposes the KDB-RRT* algorithm, which employs a three-layer optimization framework and is complemented by dedicated obstacle avoidance and collision detection mechanisms to achieve synergistic improvements in planning efficiency and trajectory quality.

The algorithm’s innovations include

(1) KD-tree-accelerated neighbor queries, reducing the computational complexity of critical retrieval steps from $O\left(N\right)$ to $O\left(N\log N\right)$.

(2) Target-biased dynamic circular sampling integrated with a bidirectional potential field guidance model, enhanced by a Sigmoid-based adaptive step size to reduce blind exploration and improve directional growth.

(3) Path morphology optimization via DP pruning and cubic B-spline smoothing, generating curvature-continuous trajectories with minimal redundant inflection points.

(4) A multi-level collision detection framework incorporates the Separating Axis Theorem (SAT) pre-check, R-tree spatial indexing, and active avoidance strategies, collectively ensuring robust collision resistance.

Extensive experiments across diverse environments (Z-shaped corridors, nested loop obstacles, and multi-obstacle maps of varying complexity) and ROS platform validation demonstrate KDB-RRT*’s superior performance: compared with state-of-the-art algorithms (Optimized RRT*, RRT*-Connect, and Informed-RRT*), the average planning time is reduced by 70.2–97.9%, path length is shortened by 4.8–21.4%, and the number of inflection points is decreased by 40–90.5%. Notably, KDB-RRT* achieved 100% success rates in high-complexity scenarios, outperforming benchmark algorithms (28–72% success rates). These results verify that KDB-RRT* effectively balances efficiency, optimality, and safety, providing a robust solution for agent path planning in unstructured environments. While validated using unmanned ground vehicles (UGVs), the algorithm’s adaptability to 2D path planning enables extension to other ground mobile agents (e.g., automated guided vehicles [AGVs] and autonomous navigation robots) requiring efficient, safe, and smooth path planning in unstructured settings.

Despite significant performance improvements in complex environments, KDB-RRT* faces challenges:

(1) Analysis has been limited to static environments without considering static–dynamic obstacle interactions.

(2) Adaptability to unstructured terrain (e.g., high-roughness surfaces) requires enhancement.

Future research will extend to dynamic obstacle avoidance and terrain adaptability optimization to improve robustness in real-world complex scenarios.

## Figures and Tables

**Table 1:** Computational complexity comparison: KDB-RRT* vs. RRT*.

Algorithmic Step	KDB-RRT*	RRT*	Optimization Mechanism
Nearest-neighbor query	$O\left(\log N\right)$	$O\left(N\right)$	KD-tree spatial partitioning (Section 3.1)
Collision detection	$O\left(\log M\right)$	$O\left(M\right)$	R-tree indexing + SAT pre-check (Section 3.5)
Rewiring operation	$O\left(\log N\right)$	$O\left(N\right)$	Optimized neighborhood retrieval (Steps 6 & 8)
Total per iteration	$O\left(\log N+\log M\right)$	$O\left(N+M\right)$	Hierarchical spatial indexing
Asymptotic	$O\left(k\left(\log N+\log M\right)\right)$	$O\left(k\left(N+M\right)\right)$	Bidirectional search + multi-layer optimization
Path pruning	$O\left(L\log L\right)$	~	Douglas–Peucker algorithm (Section 3.6.1)
Path smoothing	$O\left(L\right)$	~	Cubic B-spline fitting (Section 3.6.2)

**Table 2:** Comparative experimental data for the “Z-shaped” environment.

Algorithm	KDB-RRT*	Optimized RRT*
Average planning time/s	4.10	13.77
Average path length/cm	1258.25	1321.92
Average number of path nodes	269	827
Node utilization	54.7%	38.5%
Average distances to obstacles/cm	5.23	2.17
Minimum distances to obstacles/cm	4.69	1.36

**Table 3:** Comparative experimental data for the “Loop-shaped” environment.

Algorithm	KDB-RRT*	Optimized RRT*
Average planning time/s	0.85	3.04
Average path length/cm	646.47	721.08
Average number of path nodes	70	230
Node utilization	81.4%	67.9%
Average distances to obstacles/cm	4.36	1.89
Minimum distances to obstacles/cm	3.74	1.27

**Table 4:** Comparative experimental data of path superiority with KD-tree integration.

Algorithm	KDB-RRT*	Optimized RRT*
Average planning/s	1.13	12.95
Average path/cm	747.06	790.79
Average	97	1075
Node utilization	80.41%	41.02%
Average distances to obstacles/cm	2.56	1.63
Minimum distances to obstacles/cm	1.38	1.24

**Table 5:** Comparative experimental data on the superiority of paths introduced into the DP algorithm pruning.

Algorithm	DP Pruning	Triangle Pruning	Before Pruning
Average path length/cm	742.23	757.18	762.74
Average number of inflection points	5	11	18
Average processing time/s	0.67	1.12	~
Average distances to obstacles/cm	2.48	2.65	2.37
Minimum distances to obstacles/cm	1.65	1.71	1.62

**Table 6:** Comparative experimental data (Map 2).

Algorithm	KDB-RRT*	Optimized RRT*	RRT*-Connect	Informed-RRT*
Average planning time/s	1.11	26.68	24.34	4.86
Average path length/cm	774.89	783.60	835.66	780.14
Average number of inflection points	9	26	41	10
Average distances to obstacles/cm	2.05	1.72	1.68	2.23
Minimum distances to obstacles/cm	1.39	1.33	1.31	1.41
Planning success rate	100%	100%	100%	100%

**Table 7:** Comparative experimental data (Map 3).

Algorithm	KDB-RRT*	Optimized RRT*	RRT*-Connect	Informed-RRT*
Average planning time/s	1.17	46.76	37.16	8.26
Average path length/cm	792.48	866.32	844.52	812.37
Average number of inflection points	4	25	42	10
Average distances to obstacles/cm	4.12	1.98	1.85	4.37
Minimum distances to obstacles/cm	3.69	1.35	1.32	3.82
Planning success rate	100%	68%	72%	100%

**Table 8:** Comparative experimental data (Map 4).

Algorithm	KDB-RRT*	Optimized RRT*	RRT*-Connect	Informed-RRT*
Average planning time/s	1.96	94.77	48.57	8.58
Average path length/cm	791.88	866.13	1007.24	809.31
Average number of inflection points	9	24	59	15
Average distances to obstacles/cm	3.05	2.13	1.97	2.68
Minimum distances to obstacles/cm	2.42	1.57	1.38	2.04
Planning success rate	100%	28%	36%	98%

**Table 9:** Experimental data for intelligent agent path planning.

Algorithm	Scenario 1	Scenario 2
Average path length/m	3.23	6.72
Average planning time/s	0.78	1.64
Average traveling time of UGV/s	1.46	3.08
Average distances to obstacles/dm	3.5	2.7
Minimum distances to obstacles/dm	2.8	1.3
Whether a collision occurs	No	No

## Data Availability

Data are contained within the article.
